# Immediate Root Canal Filling

**Published:** 1911-08-15

**Authors:** P. D. Houston

**Affiliations:** Lewisburg, Tenn.


### IMMEDIATE ROOT CANAL FILLING.

BY P. D. HOUSTON, D.D.S., LEWISBURG, TENN.

Read before the Tennessee State Dental Association, May 23, 1911.

When patients came to me with aching teeth in the first ten years of my practice I said, "all right," sit down and I will extract your tooth. I did not take time to make any special examination at all. I said to myself the tooth is aching and it will do a great deal more harm to the patient than if I extract it. I was attending the Southern Dental Association at Lookout Mountain, and I heard Dr. John Story, of Dallas, Texas, talking about taking out nerves and filling the roots, and he said it could be done easily and safely and that he would guarantee it every time. I said, Doctor, what if the tooth has an abscess, would you fill it any way? He said, Yes, I would clean it out carefully and fill it, and I will guarantee it every time. I said, what will you do about it if there is pus in the pulp canal? He said, I will take a fine instrument and go clear to the apex of the tooth and clean it out. I said, if they were my patients, they wouldn't let you. He said, all right, if they didn't let me do it, I couldn't treat them. I told him when I went home I would try it, and I did, and I have been making it successful ever since.

I remember also Dr. G. G. Bass, of Clarksville, who was a member of this association, and I heard him say that by the use of cocaine he took out pulps and filled the roots of the teeth at one sitting. It is a great advantage to do this at one sitting. Suppose you have a patient who lives several miles from your office and it is not convenient for that patient to come to your office every two or three days to have the attention of the dentist. If he lived in town you could put in a treatment and he could come back again, you could fill his tooth temporarily, but in the case of the patient who lives at a distance, how much better it is to just place them in the chair and take the pulp out and fill the root and tell them to go their way rejoicing. If the tooth should happen to get a little sore, put a little iodine and aconite on it, and it will be all right in a few days. Now this is saving a great deal of the patient's time, especially if the patient is a busy man or lives away from your office a considerable distance. These are the principal reasons why it is now an accepted practice to extirpate the pulp of the tooth and fill it immediately. It can be done successfully in an hour or two and in most cases better than by a long course of treatments.

Most every dentist who does much of this kind of work has his own peculiar way of doing the work. All I can do is to tell you my procedure. Often I have had persons come into my office and tell me they want me to extract a tooth for them, that it is or has been giving them a great deal of pain. And I say, well take the chair and I'll examine it and tell you what I think about extracting it. After the examination I find a considerable cavity, with very little gum complication. I say to them, this tooth can be easily saved and be as useful to you as it ever was, and I must decline to extract such a tooth as that. If the patient is what I call a sensible one he wil say, well, I'd rather keep the tooth if the pain can be stopped and the decay arrested. If there is much tenderness in the cavity, I excavate it as much as I can without too much pain to the patient and moisten a small pledget of cotton in phenol, or some other anodyne remedy, and place it in the cavity, which generally gives present relief. I next apply the rubber dam and place on my mixing slab a little phenol and cocain and mix them well and saturate with the mix a small pledget of cotton. I next remove the anodyne treatment and excavate the cavity as much more as I can without too much pain, and I am glad if I see a little blood oozing from the bottom of the cavity. I then place the cotton I have prepared with the phenol and cocaine and warm it and place it in the cavity and press it in snugly with a suitable in trument. I then take a piece of soft vulcanite and place it over the cotton in the cavity and use my best efforts to produce gentle but firm pressure, at the same time I inform the patient that I expect it to cause some pain on the first pressure, but that it will soon become so anesthetized that the pres ure will cause no pain at all, then I extract the pulp without any, or very little, pain. I generally accomplish my object in from ten to th rty minutes. If the pulp is conjested, or in a morbid condition so that the circulation will not readily imbibe the cocain, it takes longer. If it is a very nervous patient I sometimes incorporate about one-fifth of a grain of arsenic with a little cocaine and phenol and place it in the cavity and seal it up until the next day, or longer, and when the patient returns I place the rubber dam and take out all I have put in and more too, until the pulp chamber and root canals are exposed, and then I can use freely my engine burs, broaches and excavators for opening and cleansing the canals. I use phenol freely in the chamber and root canals and follow it with iodine and hot air. I next take my root canal dryer and make it pretty hot over an alcohol lamp and insert it into the root canals as long as I think there is any moisture in them, using alternately the hot air syringe. I then force well into the root canals some "forma-percha" and place some gutta-percha on that, and in large cavities cement on that and then finish with such filling as may be indicated. Then paint the gums all around with iodine and aconite and tell the patient to repeat it if pain follows.

There is another class of cases of root filling which is more difficult than this, and while I do not think it should come under the above heading, I would like to hear it discussed. It is where the pulp of the tooth is already dead and has been indefinitely, and it is necessary to fill it to arrest the decay, or the tooth will soon be entirely ruined by decay. This class of cases have given me more trouble than the other, and I want more than one sitting of the patient in order to do the work the way it should be done. I open up these cavities with drills and excavators, using phenol all the way and not using much pressure on the instruments. I get down near the apical foramen and then place in the pulp chamber a little formaldehyde, or anything else that I may think better for the occasion, and seal it in to remain a day or two, then take it out and treat it again if I think it necessary. After I am satisfied that the sterilization is sufficient, I get the cavity as clean and dry as possible and fill it in much the same way as in the preceding case, not forgetting to paint the gums well all around with iodine.

### DISCUSSION.

Dr. M. C. Leonard: I think this is an exceedingly important subject. The immediate filling of root canals in cases where the live pulp has been extracted is, I believe, the ideal practice, if the operator uses proper aseptic precautions. If we have an infected canal to deal with, we must use good antiseptic disinfectants throughout the operation, and be sure that our instruments are sterile. If the canal is sterilized from time to time by flooding it with disinfectants, I believe at the end of the operation when the pulp tissue has all been removed we could not have a better time for its filling than just at that time, because any subsequent operation would furnish an avenue for the re-infection, which is a very good reason for not deferring it to another time. A good deal has been said about having the canals perfectly dry. I notice our essayist spoke in his essay of trying to dry the canal by a heated canal dryer heated over an alcohol lamp. I regard it as most necessary to get the canal perfectly dry. It helps in the sterilization and makes it possible to force the root filling clear to the apical opening. The canal at that point should be perfectly sterile and by using an antiseptic dressing of some kind that will go to the apex of the root, there is practically no reason for any subsequent infection. I don't see how it could occur. The canal filling should be simply a surgical dressing placed against the tissue at the end of the root. This is a surgical dressing put in perfect contact with the tissue and there can not be any infection there without your knowing, and feeling confident of it, unless the infection comes from without, because that is a wound that should heal by first intention. I think it is altogether correct to fill these canals and then to fill the cavities or place a crown on them if necessary. I have done it often at the same sitting, and without fear of subsequent trouble.

As to a case where the pulp is already devitalized, that is, of course, a different question. There is a septic condition already and the chances, it seems to me in a majority of cases, would be very much against immediate filling. I don't quite understand whether the essayist meant to fill both of these cases immediately or not, but in case this infection has extended into the apical tissues, it is usual, I think, that there would be more or less infection that would be difficult to destroy by one application of any form or kind of antiseptic that I know anything about. I think it would always, in cases of that kind, be safer to fill at a subsequent sitting, or several in fact, to be sure that the tissues have been thoroughly disinfected. I think also there is danger of too much treatment of such cases. It can easily be overdone. I think it is often overdone. There are a great many points to be considered in the treatment of cases of that kind. I must confess that I have not been overly successful in all my treatments of these cases. I find that it is often necessary to treat them for a long time, and I have suspected at times that I had done too much treating.

Another class of cases that I am hardly prepared to express an intelligent opinion on is that class where some devitalizing agents has been used. I understand there are a good many who use devitalizing agents before removing the pulp, that is in cases where they fill the canals immediately after removing the pulp. Now I don't know what success all have in using this method. I don't know that I have ever discussed this matter fully with any one who was practicing that method, but it seems to me that even in the most favorable cases, there would be considerable risk if there is any infection. If it has occurred from the devitalizing process, I think in those cases it would be wise to treat for a while and make an effort to get the canals thoroughly sterilized.

Dr. G. W. Powell: I wish to refer to that part of the paper in which the essayist referred to a tooth which had contained for a considerable time a putrescent pulp. I wish to agree with him in part, and to disagree in part. I have had a good deal of experience and I really enjoy having a putrescent pulp treatment. The best treatment I have ever used in these cases is to open into the pulp chamber, making absolutely no effort to interfere with the canal or its contents at the first sitting. I simply open the cavity and clean out the pulp chamber fairly well, and then seal into the chamber hermetically formaldehyde and cresol, and dismiss the patient for a day or two, or in some cases, several days, the time is within the discretion of the operator. Then I remove the treatment and thoroughly clean out the canal, and replace in the canal cresol and perhaps formaldehyde, and dismiss the patient for two or three days for the canal filling. I would discourage any attempt to clean out the contents of the pulp canal at the first sitting, or any attempt to fill the canals at the first sitting.

Dr. Templeton: I like the paper, as it tells me about some things I know how to do and some things that come easy, but it don't tell me how to kill the pulp in the cavity that is out of sight and almost wholly inaccessible. Another thing, he didn't say a word about was the filling of small and tortuous canals. He talked like they were all as big as gun barrels, but how are you going to fill one when you can't get a broach into it? I want to tell you how I try to do it. Instead of drying these canals as the doctor suggests, I wet them with chloroform and you can fill them much easier than when they are dry. In some teeth the canals are not large enough to introduce a stiff paste and the more liquid the easier it will be to introduce the filling material. You will find some that way and when you are filling the root canal the filling material can be led down a wet canal easier than in a dry one, it will not stick on the sides, but will follow on down to the bottom.

About immediate root filling, after you remove the pulp you frequently have hemorrhage, and if you undertake to check the hemorrhage you clot the blood in the canal and fill it sometimes half full. If you attempt its removal you are liable to start a new hemorrhage. If you undertake to fill it, you will find the bottom filled with clotted blood.

Dr. M. D. Meadows: Mr. Chairman, this subject is threshed out nearly every year before our Association, and expression of opinions each time is given, and I have come to the conclusion since having heard all these opinions that there is yet a place in the dental office for arsenic, and it is on that point I wish to speak. Pressure anesthesia, we all agree is a model surgical procedure and should be adopted in most cases. There are, perhaps, twelve single rooted teeth in the upper or lower arch, where pressure anesthesia is decidedly indicated. When the pulp is surgically removed, I agree with the essayist, that that root canal should be filled immediately, some claim while the hemorrhage is still going on. I try to stop the hemorrhage before making the filling, and I am always careful to have a surgical field to work in, and to make infection improbable.

When a patient comes from a considerable distance to have his teeth treated it is necessary to get through with the operation as quickly as possible; but for patients who are living near the office it is not necessary to keep them in the chair and for a long period or subject them to continued pain for any length of time. These patients usually come for relief, and if you can relieve them, I think it is well enough to dismiss them for another appointment. It is often difficult to make the application of pressure anesthesia to the molars. I presume that I am as skillful as the average practitioner, but I do find difficulty in applying pressure anesthesia to the teeth in the posterior part of the mouth. I have better results in these cases from sealing arsenic into the cavity with a dressing which will not become dislodged or permit the washing out of the devitalizing paste. Of course, there is danger in the use of arsenic if carelessly manipulated, but it is supposed to be intelligently used and there should be no injurious sequence. I fill immediately after the arsenic has been removed, that is to say, after removing the pulp, I scald the root canals and fill them.

Dr. Cottrell: There are a number of these root canals through which your broach will easily pass, and when you have devitalized by pressure anesthesia, there will be anesthesia of the soft tissues beyond the pulp, and it is difficult to determine when you reach the end of the root canal. There is only one means of knowing when you pass through the apical foramen and that is the pain sensation of the normal tissue. If there is any other way of knowing this I don't know what it is. If you attempt to fill these canals immediately, you have lost the benefit of the natural guiding sensation, because all this tissue is anesthetized. Did you ever do that and wonder why your teeth were sore afterwards? I do not see any way on earth to guard against that. I know it is dangerous to pass the broach through the root canal in making the filling. It is not only possible, but probable, that every time there is a root canal filled, it is infected, and very often when you are removing the pulp in your anxiety to get it all out of the root you go half an inch beyond, and you can imagine the result. If you force the instruments through in this way and use gutta-percha points for filling, you will push the point through also and have a bad result. I have taken out teeth which had gutta-percha points extending a quarter of an inch beyond the end of the root, and I would like to know if you practice immediate root filling, how you are going to obviate this difficulty. And another thing about root canal fillings that troubles me is to make a root canal filling that will last longer than six or seven or eight or ten years, I can do that easily in most cases, but is it practically impossible to make it last ten or twelve years? Dr. Black says ten years was about the lentgh of the life of a devitalized tooth. I want to call your attention to that one point, that you will carry your root canal filling beyond the end of the tooth if you fill it while the tissues are anesthetized.

Dr. Johnson: Mr. President, as I practice in a country town I try to do this operation at one sitting. I have used cocain and arsenic both, and I have had some cases that I couldn't get through very well, in molar teeth. I have been quite successful, where I have injected what is known as non-toxo in through the process, and if necessary I would force the point of my needle in sufficiently to anesthetize the pulp. I anesthetize the gum and drill through the process and make an injection of non-toxo into the nerve trunk and anesthetize it that way and can then drill through a filling, I have drilled through a filling where it had come in contact with the pulp and I have taken out the pulp at the same sitting without putting in any other treatment at all. In cases of pulpitis where the pulp is inflamed to the extent that you cannot use cocain at all, it is very satisfactory in such cases. In the case that the Doctor mentioned of moistening his canals, I fill most of my canals with a solution of carbo-formaldehyde. I wash the canals out with water and chloroform and after drying them thoroughly, I moistem them with carbo-formaldehyde, and I find that very successful.

Dr. D. M. Cattell: I want to congratulate this young man. He is a true surgeon, he has gone at this operation like a surgeon. Most dentists have gotten into the habit of doing things in one set old way, and it is the young men you will usually find who are coming to the front with some new methods and the new methods are more like the general surgical method. This leads me to what our good father started out in his remarks to say about puncturing the process to the end of the root. There are dentists who are afraid of the kinfe, they are afraid of blood, they are afraid to cut them, they can only operate on the teeth and not the parts around them. You will find all successful operators of years ago, as well as of to-day, are not afraid of cutting through the process to the end of the root, if it is necessary, which it often is. Years ago it was taught that a root canal might be filled provided the canal was sterile, and then the abscess could be treated through an artificial opening in the process, and never has there been a better way suggested, if nature was not capable of taking care of it herself. Sometimes she is and sometimes she is not.

Dr. Leonard: So far as the blood itself in the canals is concerned, if you have been aseptic in your operation the blood itself is sterile, and it need not be a hindrance to immediate root filling. By pumping in some form of antiseptic paste that is easy to force through the canal, and even in cases where it seems almost impossible to stop the blood this dressing can be made to stop the hemorrhage itself, in fact there is no better method of preventing a hemorrhage than a compress. By using an antiseptic paste of some kind the blood can be stopped by gently forcing the paste into the canal and absorbing the excess of blood by using a sufficient amount of pressure and that can be gently worked up with a smooth broach and little pellets of cotton, until you finally have the root canal filled, then the pressure of the filling itself is sufficient to prevent any considerable hemorrhage afterwards. If you are afraid that you are going to confine blood beyond the root filling, by taking a smooth broach after you have the canal almost full, and pass it through the paste while it is still soft, if there is any blood that has been left up at the apical end, it will ooze out through the canal made by the broach. Then the canal point if it has been thoroughly sterilized can be passed through that little opening, and any remaining blood there will not be sufficient to cause trouble.

As to the difficulties of applying pressure anesthesia, there is no cavity that I can imagine in a molar or bicuspid to which pressure anesthesia cannot be successfully applied. By opening the cavity in such a way that the paste can be confined directly against the pulp in the way Dr. Templeton has suggested. The paste is soft and of course pressing upon it so as to include it in the cavity and confine it against the pulp, there is no way to keep from anesthetizing the pulp. It is just as easy as applying arsenic. In fact, I think it is very much easier than to seal in perfectly an application of arsenic.

As to inserting a broach beyond the apical foramen, I don't think this makes much difference. I do not think it makes any difference if the tissues beyond have been anesthetized. We can tell pretty nearly where the end of the root is, and by using some kind of paste to fill the canal, you can force it in with just enough pressure to feel sure that you have filled the space that you have removed the pulp from. It does not make any difference if there is a little bit of it left, or a little too much tissue has been removed, if you have been aseptic in the operation. Nature will repair the damage.

As to treating abscesses through the process, as referred to by Dr. Cattell, I think it will be an excellent means of handling an abscess, if we could always feel sure that we could reach the point. You take an upper molar, for instance, that has three roots, if we fill these canals, it will be pretty hard to make an opening through the process to reach each one of the canals so that we could feel sure that there would be no infection that would not be taken care of.

A Member: The cases that cause the most difficulty are those where the pulp in the pulp chamber has become devitalized and acts as a valve and prevents the ingress of the anesthetic itself. In those cases it is often necessary to work a broach in behind it and force a little of the anesthetic in beyond the dead portion until you can reach the living portion, and gradually anesthetize it. Those cases are sometimes very painful. The others are usually treated without pain.

Dr. Henry W. Morgan: I haven't heard any mention made of the rubber dam. I want to go on record as having said that in all these cases a rubber dam should be put on first. Most of you have heard of this before. A recent advance that has been made in the treatment, or rather in the treatment of certain conditions that are met with in cases of abdominal affection in operations for appendicitis. You all remember that a few years ago our surgeons used a very extensive apparatus for carrying on irrigation and drainage for many hours. About eighty-five percent. of everybody that had drainage tubes with their peritoneum washed out that way, died; now, instead of washing it out they put in a drainage tube and let nature do the rest. I say in the treatment of many of these pulpless teeth, you had better let nature do her work. I do not agree with Dr. Cottrell and the most of my friends who want to make an application of pressure anesthesia to pulps that are inflamed, or to pulps that are partially decomposed, or in carrying the treatment into an area that is not already infected, that is taking a risk with an alveolar abscess that will exceed anything that I want to be responsible for.

Dr. Houston, closing the discussion: I said in the outset that I felt myself inadequate for the address and discussion, and of course I did not expect to exhaust the subject. One brother seemed to think that I had erred in not telling all about it. I didn't expect to tell all, I wanted to leave a little for him to tell, but I have just a little more to say in regard to the work. Dr. Morgan said nobody had said anything about the rubber dam being used. I know that was in my paper, if I did not say it, I skipped it.

We have cases sometimes where there is sever pulpitis, making treatment of any kind difficult and unsatisfactory. I had one just a few days ago, but I told the lady that if she could stand it until the cocain could have effect on the pulp, I thought I could take it out practically without pain. I worked on it something like an hour trying to anesthetize the pulp and did not finally succeed and then I used my arsenic, I put a little piece of arsenic, cocain and phenol on a piece of cotton, inserted it in the cavity and sealed it in and told her to come back next day. When she came back I put on the rubber dam and took out the treatment. I was very anxious to find out whether that pulp was anesthetized or devitalized. I began to excavate down into it, and when I had most of the pulp out of the pulp chamber it became very tender again. I applied the cocain again and used the pressure anesthesia, which gave some little pain at first, but after five or ten minutes, I pressed as hard as I wanted to press and there was no pain. I took out the cocain and found that the pulp was anesthetized almost to the apical foramen. I didn't care about going any deeper into the canal, so I put in a little more cocain and used pressure anesthesia, and found there was no pain, and then after properly sterilizing the canal I filled it. I have heard no complaint from it since, and that was quite a while ago. I have no idea that there has been any pain since.

Some one said something about the blood being in the way. That does happen sometimes, and I do not like to fill a tooth unless I can arrest the bleeding from the end of the root canal, and I hardly ever do. I use hematics sufficiently to get the blood stopped until I can fill the root canal. I do not like to fill it while it is bleeding. I have known such things to be done, and successfully done too, but I do not like to do it myself if I can help it.

